# Enhancing Cancer Screening and Early Diagnosis in India: Overcoming Challenges and Leveraging Emerging Technologies

**DOI:** 10.7759/cureus.78808

**Published:** 2025-02-10

**Authors:** V Mangayarkarasi, Elantamilan Durairaj, Vijaya Ramanathan

**Affiliations:** 1 Microbiology, All India Institute of Medical Sciences, Madurai, Madurai, IND; 2 Anatomy, All India Institute of Medical Sciences, Madurai, Madurai, IND

**Keywords:** artificial intelligence in radiology, barriers, breast cancer, cancer prevention and control, cancer screening test, cervical cancer, digital pathology, early detection, national health systems, next generation sequencing (ngs)

## Abstract

This review addresses the significant challenges and technological developments in cancer screening and early diagnosis in the context of India’s diverse and resource-constrained healthcare landscape. Selected cancers like breast, cervical, oral, lung, and colorectal cancers are focused on, and established screening methods such as clinical breast examination (CBE), mammography, visual inspection with acetic acid (VIA), HPV DNA testing, and oral visual inspection (OVI) are reviewed. These are cost-effective strategies that are proven to reduce mortality. However, they face systemic barriers, including low awareness, socio-cultural stigma, and discontinuous healthcare access.

Emerging technologies in cancer screening like liquid biopsy (detecting circulating tumor DNA), artificial intelligence (AI)-driven imaging (enhancing radiological accuracy), next-generation sequencing (identifying genetic mutations), and methylation-based ctDNA analysis (epigenetic profiling) are considered to be transformative in cancer management. Digital pathology and telemedicine are also found to improve diagnostic precision and rural/remote outreach. However, high costs, technical complexity, and limited validation in Indian settings are the major challenges that hinder their widespread adoption.

The review emphasizes the need for culturally tailored awareness campaigns, integration of screening with the already existing public health programs, and increased investments in indigenous research to address genetic and environmental risk factors. It specifically advocates for strengthening the primary healthcare infrastructure, training community health workers, and leveraging mobile screening units to bridge urban-rural disparities. A combination of scalable low-resource methods and strategic adoption of emerging technologies can help in mitigating India’s growing cancer burden. This aligns with global targets to reduce premature non-communicable disease (NCD) mortality by 2030. This synthesis of evidence-based practices and innovative strategies offers a roadmap for policymakers and stakeholders to enhance equitable cancer care delivery nationwide.

## Introduction and background

Globally, cancer is the second leading cause of death. Low- and middle-income countries contribute majorly to this mortality data. It causes immense suffering for patients as well as their families. Apart from the physical illness, it also results in a significant financial burden for all the stakeholders involved, causing mental distress and social stigma. Responding to this global health challenge, the World Health Assembly, during its 70th session (WHA70.12, May 31, 2017), urged all the nations and the World Health Organization (WHO) to enhance efforts to improve the quality of life and well-being of everyone. The objective of this is to decrease premature deaths occurring due to non-communicable diseases (NCDs), which include cancer, at least by a third before reaching 2030 [1].

It is evident that certain populations face disparities in getting exposed to multiple risk factors and lack adequate accessibility to screening and early diagnosis. Also, lack of appropriate care in a timely manner is beyond reach for these populations, resulting in poor cancer outcomes. This necessitates understanding the fact that different cancer prevention strategies are necessary for different populations of cancer patients (e.g., children, women, adolescents, elderly, etc.) and also realizing that a reduction in risk can prevent approximately 50% of all cancers in these populations [1].

India, with its dense population and diverse religious and cultural practices, faces unique challenges in implementing cancer prevention and care strategies [2-4]. Cultural barriers include communal stigma and misconceptions, traditional beliefs on reliance on traditional/alternative medicines, and gender bias. Logistic barriers include geographical disparities, economic constraints, and lack of education and awareness [5]. This article aims to present cancer screening methods for early detection of certain common cancers, including breast, lung, prostate, cervical, and oral cancers while reviewing various barriers to this seen in the Indian population. To overcome these barriers, the Indian health system must be streamlined, following practices that can be adapted from those in developed countries. Certain practices like implementing robust Electronic Health Record (EHR) systems, expanding telemedicine services, promoting public-private partnerships (PPP), integrating digital initiatives, and assuring universal health coverage can be effectively adapted to the Indian healthcare system [6].

With a reported 1.2 million incident cancer cases in 2018, India positions itself in third place in the world with respect to the incidence behind the United States and China. Also, second in terms of an estimated 785,000 fatalities from the disease [7]. Cancer being a serious public health concern reiterates how important it is for the health systems to be ready to handle the increasing cancer burden. Guaranteeing timely and accurate diagnosis with proper treatment is very essential to handle the challenges posed by cancer. In addition to individual impact, the increasing cancer rate also contributes to increasing financial, emotional, and physical burdens on the families, community, and national health systems [8]. Tamil Nadu in India had an overall cancer incidence rate of 84.2 per 100,000 people. Different regions of the state had varying incidence rates, with Chennai, the capital city, showing the highest rates, especially for women (152.4 per 100,000) and males (129.3 per 100,000) [9].

The purpose of this review is to address cancer, a significant public health problem in India, where the disease burden is quite high, which is further complicated due to delayed diagnosis. The research gap identified is the knowledge of specific challenges in implementing cancer screening and early detection programs in India and the lack of tailor-made solutions to improve outcomes in this context. This paper tries to emphasize the importance of combining contemporary screening methods with newer technologies while addressing the existing barriers to access and awareness. Selected cancers were focused on because these were the most common cancers among males and females as per recent Indian data. In India, lung cancer accounted for most cancers in males (10.6%), followed by oral (8.4%) and prostate (6.1%). Among females, breast cancer was the commonest (28.8%), followed by cervical cancer (10.6%) [10]. By highlighting the current screening methods and available technological advances, along with the need for improved healthcare access and information, the review offers comprehensive recommendations for decreasing cancer mortality and improving overall cancer care in India. Key recommendations of this review include strengthening primary healthcare services, addressing the socio-cultural barriers, integrating cancer screening with existing programs, investing in indigenous research, leveraging technology, and improving access to affordable care.

## Review

Cancer: early diagnosis and screening

The burden of cancer is rising in low-income and middle-income countries (LMICs), where an estimated 65% of global cancer-related deaths occur annually [11]. The probability of successful treatment is greatly increased by early detection of cancer. Screening and early diagnosis are the two components of early cancer detection. Early diagnosis aims to discover patients with symptoms as soon as feasible, whereas screening tests are conducted on healthy persons to find cancer patients before symptoms show up [12].

Distinguishing cancer screening from early diagnosis

Making a diagnosis early requires early patient presentation, as soon as the first symptoms appear. It is applicable to all forms of cancer. Contrarily, screening is applicable for the whole population but only useful for a small group of cancer types, including breast, colorectal, and cervical cancers, which together account for about 30% of cancer incidence in the European (WHO) region. With minimally invasive surgical techniques, cervical cancer can be completely cured when it has presented in a precancerous stage of the illness, thanks to screening. However, similar results may not be applicable to colorectal cancer screening or breast cancer screening [12].

Cancer screening in India

Over the past several decades, India has seen a rise in the incidence of cancer. The cancer incidence rate increased by 34.94% in 2021 when compared to 1991. The mortality rate increased from 41.39 to 60.44 per 100,000 population during the same period. This represents a 46% increase in the last three decades [13]. Of all the cancers that may be easily recognized in their early stages and are avoidable, the most prevalent ones in India are oral, breast, and cervical cancers [14]. Population-level screening is very beneficial to the public when it is coordinated and prepared for, but when it is not, expenses can go up, and benefits might be diminished [11]. Currently, WHO guidelines are available for LMIC settings for screening oral, breast, and cervical cancers. However, there are significant disparities and variations in the accessibility and availability of healthcare resources, which present challenges to their adoption and use. Key disparities include financial disparities, availability of trained manpower, and access and referral facilities [15].

Thus, as part of the National Programme for Prevention and Control of Non-communicable Diseases (NP-NCD), the Indian government introduced the operational guidelines for population-based cancer screening of the three major malignancies in 2016 [16,17]. The population aged 30 to 65 will be eligible for screening by the criteria, and the auxiliary nurse midwife (ANM) or nurse assigned to the subcenter will conduct the screening [18].

Even though our government has established broad program-based guidelines, they present particular difficulties like administrative costs, workforce training, screening facility accessibility, follow-up, and sufficient referral connections for a confirmation diagnosis and treatment afterward. Since health is a state matter in our nation, the evolution of public health care systems differs from state to state, as do the health resources allotted to the fight against cancer [15].

Screening approaches

There are well-established, low-cost screening methods used across the world to lower the death and morbidity rates from oral, cervical, and breast cancer. Commonly employed methods for a few selected cancers are briefed below:

Cervical Cancer

The prevention and early diagnosis of cervical cancer involve a range of interventions, including HPV vaccines and screening techniques like visual inspection with acetic acid or Lugol's iodine (VIA/VILI), Papanicolaou test (Pap test/smear), and HPV DNA testing [19,20]. A single-visit screen and treat approach is made possible by VIA's primary screening, which produces timely results when paired with cryotherapy facilities. The recommendations listed in Table 1 can enhance early diagnosis and screening [15].

**Table 1:** Recommendations to enhance screening and early diagnosis ANM: auxiliary nurse midwife; VIA: visual inspection with acetic acid

	List of recommendations
1	Paramedical staff with training, such as ANMs, can conduct VIA up to the subcenter level
2	Confirmation can be done at primary health centers (PHC), Community Health Centers (CHC), and district hospitals (DH) for those who turn positive in screening, utilizing the available tests, such as cytology and colposcopy-guided biopsy
3	The following locations will do biopsies: DH, PHC, and CHC
4	The "see and treat" approach applies to PHC and higher levels
5	Precancerous lesion therapy, biopsies, and colonoscopies are at District hospitals

Breast Cancer

Mammography is one of the most commonly used screening techniques, and it is found to be beneficial for women in the age range of 40 to 74. The advantages of clinical breast inspection and breast self-examination are still unknown despite research on the subject. Tomosynthesis, magnetic resonance imaging, ultrasound, and molecular breast imaging are among the technologies being assessed; these are frequently employed in addition to mammography. Clinical breast examinations (CBEs) performed by trained healthcare workers are an inexpensive way to screen for breast cancer in low- and middle-income countries. Given the typical tumor size at diagnosis, the late diagnostic stage, and the socioeconomic circumstances in developing nations like India, CBE may be a helpful method for breast cancer screening as a first modality [19,20]. The recommendations listed below can enhance early diagnosis and screening: If the healthcare system meets the requirements for implementing an organized program, the WHO puts forward organized, population-based screening programs with mammography for women aged 40 to 69 who are asymptomatic and have an average risk for breast cancer in settings with optimal resources. A two-year screening interval, however, in environments with inadequate resources, low-cost screening techniques like CBE appear to be a promising strategy that may be used once the required data from ongoing research are available [15].

Oral Cancer

Precancerous lesions can be identified early through self-examination, visual inspection of the oral cavity at routine check-ups, or screening check-ups by medical professionals such as dentists or physicians. The early detection and diagnosis of precancerous lesions and malignancies in the oral cavity are accomplished by the use of oral and visual inspection, exfoliative cytology, vital tissue staining (toluidine blue, methylene blue), visualization adjuncts (ViziLite Plus with TBlue, ViziLite, etc.), and OralCDx brush biopsy [19,20]. The sensitivity and specificity of these tools are mentioned in Table 2 [21-23].

**Table 2:** Sensitivity and specificity of screening methods used for oral cancer

Screening method	Sensitivity (%)	Specificity (%)
Oral visual inspection	70-80	60-70
Exfoliative cytology	60-70	70-80
Vital tissue staining (TBlue)	75-85	65-75
ViziLite Plus with TBlue	80-90	70-80
OralCDx Brush Biopsy	85-95	75-85

Impact of screening on overall mortality of cancer patients

Cervical Cancer

According to a cervical cancer modeling study, 3,824,700 cases of the disease and 2,878,300 deaths were expected to occur in India between 2017 and 2026 for women in the 30- to 34-year-old age range who did not receive screening. These numbers translate to lifetime risks of 2.5% and 1.9%, respectively, of the 15 crore women in this population who were alive when they were first eligible for screening. In low-resource areas with a high cervical cancer burden, even brief delays in instituting organized screening programs will result in significant morbidity and mortality. This modeling study assumed a population coverage of 10% (in both immediate and delayed implementation) with a 10% loss to follow-up in the age group of 30-34 years [24].

Breast Cancer

In Mumbai, India, a study examining the impact of CBE screening on mortality from breast cancer assessed in a prospective randomized controlled trial (RCT) that followed up for 20 years found that CBEs performed every two years significantly reduced the stage of breast cancer at diagnosis. This trial included 151,538 women in the age group of 35-64 selected from 20 clusters in Mumbai. They were randomly assigned to two arms. One arm received four rounds of CBE by trained healthcare workers every two years, followed by five rounds of active surveillance every two years. The arm received only eight rounds of active surveillance every two years. The examinations were done by primary health workers, which resulted in a 15% overall reduction in breast cancer mortality, which was not statistically significant. However, women aged 50 and older saw a substantial 30% reduction in mortality [25] when compared to women who did not attend screening exams. This difference could be due to undetermined biological factors. Women who attended screening mammography examinations at least in one of their last two invitations before making a diagnosis of breast cancer showed a significantly lower risk of mortality associated with breast cancer; women who participated in both screening appointments showed the most significant benefit (of about 49% reduced risk). Also, 295,490,91 women in nine Swedish counties who were eligible for screening mammography between 1992 and 2016 were included in this prospective research [26]. However, the direct applicability of this study needs exploration in the Indian context.

Oral Cancer

The mortality rate due to oral cancer among tobacco or alcohol users, or both, was significantly reduced by 34% in a community-based cluster randomized controlled intervention trial using Oral Visual Inspection (OVI) in Trivandrum district, Kerala, India. This study enrolled more than 96,000 subjects and followed up for 15 years. Furthermore, there was a 38% reduction in oral cancer incidence and an 81% reduction in oral cancer mortality in the participants who completed all the rounds of screening in a randomized experiment done in India [27,28].

Other Cancers

GB cancer: A study suggested that all cholecystectomy specimens in India and other countries with comparatively higher incidences of gallbladder carcinoma be sent for histopathology examination. Alternatively, in resource-constrained settings, the authors have suggested selective histopathological examination of the cholecystectomy specimens to reduce cost, taking into account a few parameters like clinical presentation, intraoperative findings, etc., but this needs further studies. Only histopathological studies can identify gallbladder cancer at an early, potentially treatable stage. This early diagnosis resulted in a 72% increase in the detection of gallbladder carcinoma at an early stage, which is significant for a malignant disease with very high mortality [29]. Median survival rates were at least five times higher among those who had an early diagnosis of gallbladder cancer [30].

Pancreatic cancer: Pancreatic cancer is mostly diagnosed at an incurable stage. In individuals at higher risk, annual and one‐time screening improved life expectancy, even with a poor screening test [31].

Though we have a reasonable amount of screening tests, their efficacy and accessibility are significantly impacted by various barriers. The following discussion delineates the common hindrances that impede the widespread adoption and effective implementation of cancer screening programs. By understanding and addressing these barriers, we can strategize better and act on interventions that enhance the reach and efficiency of these cancer screening initiatives.

Barriers to screening and early detection of cancer in India

**Figure 1:**
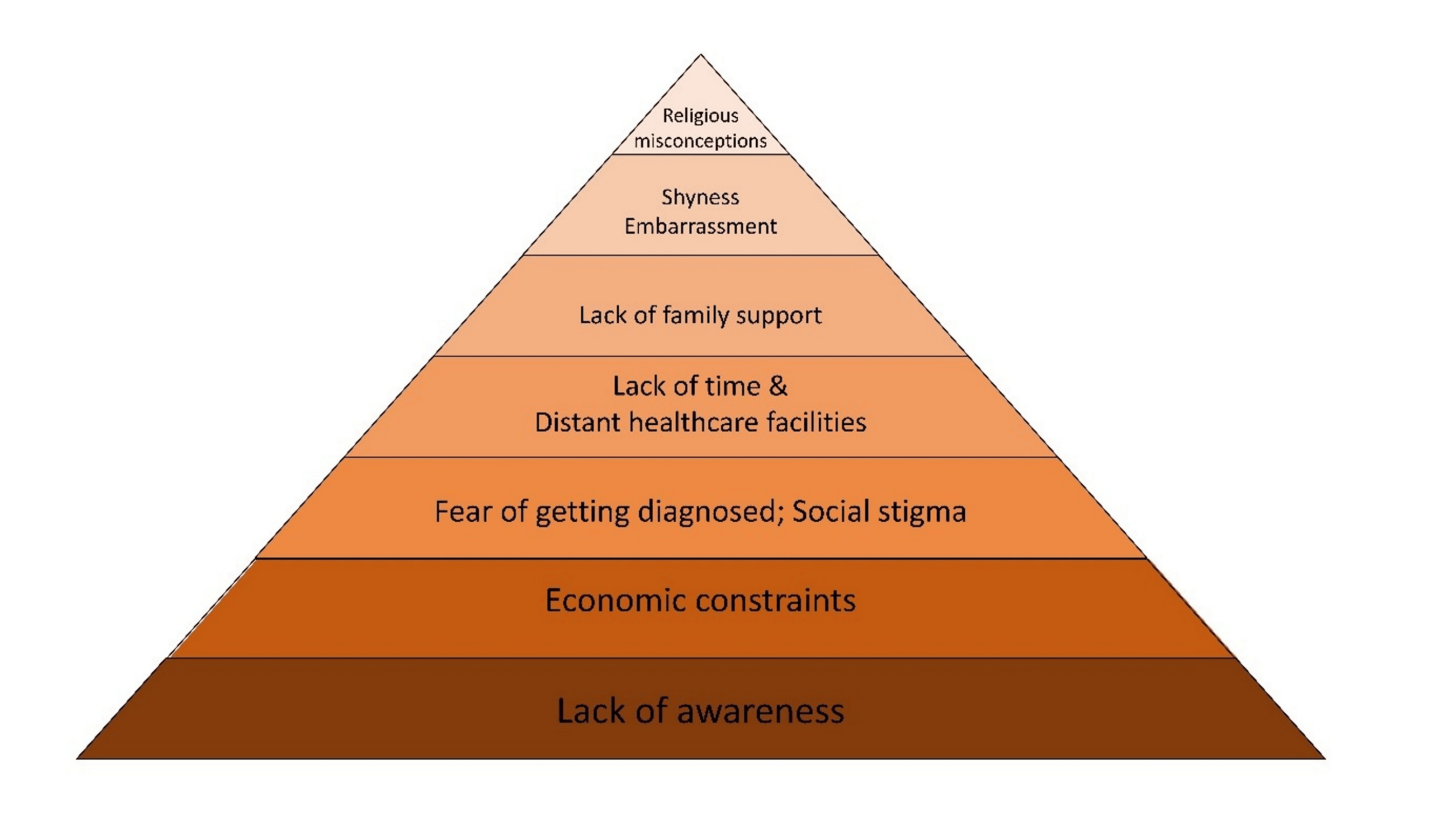
Barrier pyramid: list of barriers to cancer screening Credit: Image created by the authors

An assessment of the barriers among Indian rural women showed that reasons for not undergoing screening were as follows: 84% were not aware of such a program, 4% had economic constraints, 4% couldn’t get time, 1% were aware but did not know where and whom to approach, 1% felt shy and embarrassed, 1% said the healthcare facility was far from their residence, and 0.4% said that there was no family support and was not permitted. Interestingly, 4% did not undergo screening because of the fear of getting a cancer diagnosis [32]. Also, misguided belief in religion and fate, loss of wages, etc., had an important role in reduced accession to such screening programs [33]. Though the proportion is different, similar factors have impacted screening programs even in urban populations [34]. The various factors identified in these studies are shown in a pyramid chart (Figure 1) based on the amount of significance they play as a barrier.

Psychosocial issues, such as anxiety about the screening process and fear of receiving a cancer diagnosis, were identified to be the main obstacles to participating in screening programs in another study conducted in Tamil Nadu. A significant obstacle to participation, according to many participants, was their dread of receiving a cancer diagnosis and its ramifications. It was also connected to the misconception that cancer is contagious, the stigma attached to the disease (that it is terminal), and fear of discrimination from family and society [35]. Other contributing reasons include cultural and a lack of information, in addition to financial difficulties and issues pertaining to the healthcare system. Few women expressed concern about the high costs associated with extended therapy and more sessions. Government-mandated screening programs, awareness efforts, and incentives facilitate the increase in women's screening uptake [35,36].

The following are the obstacles that a feasibility health system study encountered when attempting to use community health workers (CHWs) to do population-based common cancer screening: The act of community motivation to screen was one of the challenging problems CHWs had to deal with. It was difficult for them not to have any cancer-related symptoms, and it is vital to inform them before planning screening events. The neighborhood residents had not yet come to terms with screening as a preventive step and had not given hospitalization any thought. Increasing knowledge contributed to the community's acceptance of the procedure. The community and CHWs had an innate fear of cancer, which made the screening process difficult. Similar feelings of terror were found in other research carried out in India and overseas, where cancer was associated with fear and death. It has been observed that pessimistic, fatalistic attitudes impede people's adoption of cancer screening and prevention measures. To participate in the study, CHWs faced challenges from unsupportive supervisors, competition for public health projects, and unfounded rumors in the community. As a result, they needed the master trainers' guidance, time management skills, and assurance [18].

In an analysis of a highly educated cohort without affordability and accessibility issues, socio-cultural barriers were found to play a major role despite high rates of awareness. They were prevented from participating in screening programs by a variety of factors, including the social stigma of being called a "cancer patient" by friends and neighbors, fear of loneliness, concerns about losing their entire breast if a lump or cancer was discovered, etc. [37].

Undesirable effects of screening programs

Even with appropriate implementation, screening programs have unintended consequences that should be addressed and include:

1. Erroneously positive screening test findings that cause patients to become anxious and undergo invasive diagnostic procedures and extra testing.

2. Erroneously negative screening test findings that cause false comfort and postpone the presentation or diagnosis of symptoms until they manifest. This would require periodic re-screening to minimize this risk.

3. Overdiagnosis and treatment of preclinical malignancies that may not have presented a significant risk to health or generated symptoms, leading to unnecessary and harmful therapy for the patient [12].

Strategies to be adopted by India for cancer prevention and control

India should adopt various strategies for cancer prevention and control, including

1. Introduction of new cancer screening devices based on investment in innovation for cancer detection. These devices or techniques should be adaptable in resource-limited rural settings, as nearly two-thirds of the Indian population resides in rural areas.

2. To improve patient access, it is imperative that barriers to cancer prevention, screening, diagnosis, and treatment, including surgery, be removed. Additionally, national health systems and cooperation with international agencies, particularly in the area of human resources, need to be strengthened.

3. A roadmap outlining the commitments to be taken up by heads of state to combat cancer needs to be developed. This includes promoting more awareness campaigns, providing financial and infrastructure commitments, strengthening comprehensive primary health care, etc. This should be coordinated among the stakeholders, including government ministries, healthcare bodies, non-governmental organizations (NGOs), international organizations, and the public community.

4. A monitoring tool with international cooperation to observe and communicate the cancer screening process, the detection rate, and further action plans.

5. Household surveys, population-based cancer registries, and similar health information systems are to be used to collect population-based cancer incidence and death data stratified for all age groups and cancer types, including imbalance assessments. Establishing mobile screening units for common cancers like breast, cervical, and oral cancers to reach remote and underserved populations.

6. Integrating cancer screening programs with already existing public health programs and strengthening the telemedicine infrastructure. Also, targeted approaches focusing on specific populations/specific cancers can be helpful. Some targeted programs like Detect Early and Save Her/Him (DESH) in Assam, India, the cervical cancer screening program in Australia, and the National Breast and Cervical Cancer Early Detection Program (NBCCEDP) in the United States were found to be effective.

7. Implementation of the time-bound national commitments for a specific duration outlined in the cancer screening outcome document in various regions of the nation.

8. Implementing the developed national cancer control programs effectively so that they are cost-effective, inclusive of all age groups, have sufficient resources, oversight, and responsibility, and look for cost- and resource-saving opportunities with other health initiatives.

9. Developing culturally sensitive strategies addressing the socio-cultural barriers prevalent in India.

10. Developing and implementing evidence-based protocols and well-structured awareness programs for cancer screening management in children and adults.

11. To establish centers of excellence for cancer screening and early detection and cooperate by bolstering regional and subregional collaborations and networks as needed. Strengthening the National Cancer Registry to record data from diverse populations of India.

12. Encourage recommendations that assist in clinical decision-making and timely referrals, based on the efficient, secure, and economical use of cancer diagnostics and treatment services in the target population's immediate vicinity. Developing or modifying resource-stratified, step-by-step instructions and toolkits to create and carry out all-encompassing cancer prevention and control initiatives. Similar initiatives by the National Cancer Grid, which is a network of cancer care institutions in India, facilitate the exchange of knowledge, expertise, and resources among member institutions.

13. Intense and specialized training for healthcare professionals with education modules, protocols for mock drills, and skill-based demonstrations about cancer screening procedures to inform the targeted population. The key competencies include clinical and technical knowledge, communication skills, cultural and linguistic skills, system navigation, behavior change, and motivational skills, etc. [38,39].

14. Investing in indigenous research focusing on factors specific to the Indian population. This would help us form tailor-made policies specific to the Indian setting, considering the genetic diversities, socioeconomic and environmental factors, and cultural and dietary practices specific to this region.

15. Establishing a national cancer survivorship program addressing the unique needs of Indian cancer survivors, including social reintegration and economic rehabilitation.

16. Middle-income countries planning to implement cancer screening programs should begin on a limited geographical scale before expanding. It is more practical and effective to target 80% of the intended population, screening them once or twice in their lifetime with a sensitive test. Managerial guidelines are now available to help in planning and implementing appropriate programs in low-resource settings [40,41].

Strategies for a program executive team focused on cancer screening and early detection

Providing the executive team in charge of cancer screening and early detection with a comprehensive technical report requires examining pricing strategies, including transparency, and their impact on the affordability and accessibility of diagnostic tests for cancer prevention and treatment. This should include reviewing benefits and adverse effects comprehensively, promoting investments in cancer research and development, periodically evaluating the innovation measures, examining the price and value relationship, picking up the financial gaps existing in cancer research in a timely manner, and exploring the options for increasing the affordability and accessibility of these measures.

Screening tests include the following: 1) physical examination and medical history; 2) laboratory tests for markers, by-products, and supporting evidence; 3) imaging; and 4) genetic tests that specifically look for chromosomal or genetic alterations.

Cancer Screening Tests

Cancer screening tests are available in referral hospitals in various regions of India [42].

Breast cancer screening: Mammography for women at risk, especially those with family history, personal history, radiation exposure, ethnicity, and genetic factors [43].

Breast MRI: Women with a deleterious mutation in either the BRCA1 or BRCA2 gene are frequently referred for a breast MRI imaging test; these mutations increase the risk of developing breast cancer in addition to other cancers.

Cervical cancer screening: Pap smears and human papillomavirus (HPV) DNA tests, which can be done separately or in combination.

Colorectal cancer screening: Sigmoidoscopy, colonoscopy, and stool testing (high-sensitivity fecal occult blood tests and stool DNA tests) are found to lower the chance of mortality from colorectal cancer.

Lung cancer screening: Low-dose helical computed tomography (CT) scans can lower the number of lung cancer deaths among heavy smokers.

Alpha-fetoprotein blood test: When attempting to identify individuals at high risk of liver cancer at an early stage, the alpha-fetoprotein blood test is occasionally used with hepatic ultrasound imaging.

CA-125 test: When ovarian cancer is suspected, especially in women who are at a higher risk, an early detection method such as a transvaginal ultrasound combined with the CA-125 blood test may be utilized. However, the low sensitivity and specificity of this test should also be considered while interpreting the results [44].

Prostate-specific antigen (PSA) test: Prostate cancer screening with PSA blood test, along with a digital rectal exam.

Transvaginal ultrasound: The transvaginal ultrasound imaging test is sometimes performed on women who have an increased risk of endometrial cancer (Lynch syndrome) or ovarian cancer (due to harmful BRCA1 or BRCA2 gene mutations). The test can produce images of the uterus and ovaries. However, it hasn't been demonstrated to lower cancer-related mortality.

Advanced multi-cancer detection (MCD) tests available in developed countries

Tests such as MCD look for biological signals secreted by cancer cells in various body fluids. These signals are referred to as tumor markers or biomarkers. These can assist in the timely identification of various cancer types depending on the signals being analyzed. RCTs are necessary to determine the utility of such MCD tests for cancer screening in individuals without symptoms.

Virtual Colonoscopy

The colon and rectum can be viewed from outside the body via a virtual colonoscopy. If this test is the only one a person finds acceptable for colorectal cancer screening. In that case, it may be advised that even though it has not been demonstrated to lower deaths from the disease, it may indicate potential issues outside the colon that require more investigation.

Emerging modalities for cancer screening

New technological developments have brought in a new era of accuracy and effectiveness in cancer screening, and early diagnosis is still essential to improving patient outcomes. With the introduction of cutting-edge techniques and diagnostic platforms, the field of cancer screening is experiencing an enormous transformation. Through technologies such as liquid biopsy, AI-driven imaging, next-generation sequencing (NGS), and multi-omics techniques, physicians are gaining unparalleled insights into the complex molecular biology of cancer.

Liquid Biopsy

A significant breakthrough in cancer screening has been the introduction of liquid biopsy [45]. This non-invasive procedure includes detecting circulating tumor cells (CTCs), cell-free DNA (cfDNA), and other indicators found in blood or other body fluids [46]. Liquid biopsy makes identifying genetic cancer easier and enables real-time tumor dynamics monitoring [47]. Liquid biopsy, which can be used to identify specific genetic changes (e.g., BRAF/epidermal growth factor receptor (EGFR) genes), has been found to be an important tool for developing targeted therapy [48]. It also provides information about minimal residual disease that helps to evaluate treatment response and monitor recurrence.

Next-Generation Sequencing

The NGS technology has revamped cancer diagnostics to a greater extent [49]. NGS that sequences entire exomes or certain genetic panels helps in identifying all the genetic alterations that are linked to cancer in a timely manner [50]. Specific treatment methods and customized treatment plans can be done using this data. Identifying germline mutations in cancers that are inherited can help with screening and performing preventive interventions in vulnerable populations [51]. NGS can be very useful in identifying these mutations, and it also plays a vital role in precision oncology since it helps in deciding the choice of treatments based on the distinct genetic makeup of the cancer [52].

Artificial Intelligence in Radiology

Advancements in artificial intelligence (AI) have transformed the medical imaging field by providing rapid and accurate data interpretation [53]. When used for MRI, CT, and mammography pictures, AI algorithms are capable of identifying even very minute abnormalities in CT/MRI that can reveal the existence of tumors [54]. By decreasing the time required for image interpretation, algorithms like these increase diagnosis accuracy and boost radiologists' productivity. AI-powered image analysis has tremendous potential for the early detection of breast cancer. Through prompt intervention and increased overall survival rates, computer-aided detection systems can detect microcalcifications and other early indicators of breast cancer. However, the current limitations, such as limited validation, robustness, and ethical issues, should be addressed [55].

Digital Pathology

Histological samples can be viewed and analyzed remotely thanks to digital pathology, which digitizes conventional glass slides. Active institutional support, spatial requirements, trained manpower, digital storage capacities, server setups with backups, throughput, and integration with existing systems can pose challenges to transformation into digital services [56]. This methodology promotes inter-pathologist collaboration and allows AI algorithms to be applied for computer-aided diagnosis [57]. Diagnostic reports can be produced more quickly when pathologists use digital pathology [58]. In addition, AI systems can help detect minute morphological alterations that point to early-stage cancer, thereby raising the sensitivity and specificity of pathological diagnosis [59].

Methylation of Circulating Tumor DNA

Beyond conventional liquid biopsy, circulating tumor DNA (ctDNA) study focuses on epigenetic changes, particularly DNA methylation patterns [60]. Since aberrant DNA methylation is a defining feature of cancer, it is possible to identify cancer-related alterations by analyzing these epigenetic fingerprints in ctDNA [61]. This approach holds great promise for tracking therapy response and detecting cancer in its early stages. However, the development of sensitive and specific diagnostic tests faces hindrances from the heterogeneity of epigenetic events and the overlapping of these changes that occur in different cancers. Also, these tests are technically demanding and currently not cost-effective for clinical laboratories [61]. The discovery of methylation patterns linked to various cancer types makes a specific and sensitive screening method possible [62].

## Conclusions

The importance of cancer screening and early detection has been reviewed, addressing the barriers and potential strategies. Implementing new technologies, improving access to care, strengthening national health systems, the need for a roadmap and monitoring tools, and investing in training and research could help in breaking the barriers. Further translational and implemental research is required to identify and address the gaps. Sustainable domestic human and financial resources must be mobilized to ensure that developing nations like India have equitable and reasonably priced access to cancer care. Additionally, voluntary and creative finance options should be considered to support cancer control. Government bodies, research organizations, foreign agencies, NGOs, etc., should work together in the effective utilization of resources. Policy interventions in the form of subsidized investigations and therapy, scaling vaccination campaigns, tax incentives for investments in research, regulatory changes, etc., can be very helpful. Healthcare workers should receive extensive training that includes education modules, protocols for simulated exercises, and skill-based learning about cancer screening techniques to inform the targeted population. The key competencies include clinical and technical knowledge, communication skills, cultural and linguistic skills, system navigation, behavior change, motivational skills, etc.

Effective implementation of cancer prevention and control services involving early detection and screening requires collaborations between the community and government, along with building on the work of patient organizations and NGOs involved in health issues. Strengthening the ability of the various states of India requires the country to support the adoption of economically viable interventions and nationally tailored early cancer detection models. It must collaborate with global partners to standardize and share the technical expertise. Nations with constrained resources should begin on a limited geographical scale before expanding, and available specific managerial guidelines should be utilized. The targeted approach in cancer screening is particularly relevant in the public health context since it offers practical guidance for healthcare practitioners, policymakers, and researchers working to improve cancer outcomes while lowering the prevalence of cancer in India.
